# Dysphagia and geriatric syndromes in older patients admitted to an intermediate care unit: prospective observational study

**DOI:** 10.1007/s40520-025-02950-8

**Published:** 2025-03-17

**Authors:** Francesca Dini, Stefania Mancini, Alessia Girelli, Daniela Perelli Ercolini, Alessandro Reggiani, Yanely Sarduy Alonso, Marco Inzitari, Giuseppe Bellelli, Alessandra Marengoni, Simona Gentile, Alessandro Morandi

**Affiliations:** 1 Intermediate Care and Rehabilitation, Azienda Speciale “Cremona Solidale”, Cremona, Italy; 2 REFiT Bcn research group, Vall d’Hebron Institute of Research (VHIR) and Parc Sanitari Pere Virgili, Barcelona, Catalonia Spain; 3 Acute Geriatric Unit, IRCCS Foundation San Gerardo, Monza, Italy; 4 School of Medicine and Surgery, University of Milano-Bicocca, Milan, Italy; 5 Department of Clinical and Experimental Sciences, University of Brescia, Brescia, Italy

**Keywords:** Geriatric syndrome, Dysphagia, Delirium, Older adults, Intermediate care, Rehabilitation

## Abstract

**Background:**

Dysphagia is a geriatric syndrome often unrecognized or underestimated, and there is a lack of studies in a heterogeneous population in intermediate care (IC) services. This study aims to describe the prevalence of dysphagia and its association with geriatric syndromes in older patients in IC.

**Methods:**

Prospective cohort study of patients 70 years and older admitted to an IC unit. At admission, the severity of the clinical conditions, comorbidity, delirium, frailty, sarcopenia, nutritional status, and medications were assessed. Each patient was evaluated with the 3-OZ test, and dysphagia was confirmed by a speech therapy consultation. Two multivariable logistic regression models were used to evaluate the association of dysphagia at admission with geriatric syndromes (model 1), along with the severity of illness and admission diagnosis (model 2).

**Results:**

A total of 455 patients were included. The prevalence of dysphagia was 10% and there was a high prevalence of mild-moderate dysphagia in patients with cognitive impairment and moderate risk of malnutrition. In the univariate analysis, an association was found between dysphagia and sarcopenia, malnutrition, and use of antipsychotics. In Model 1, higher odds of dysphagia were associated with the severity of comorbidity (Odds Ratio 6.49, 95% Confidence Interval: 2.02–20.78), and cognitive impairment (OR 0.91, 95% CI: 0.88–10.62); in Model 2, the severity of clinical conditions-NEWS2 (OR 1.61, 95% CI: 1.23–2.13) was associated with dysphagia, besides the severity of comorbidity and cognitive impairment. In a subset of 300 patients, delirium was also associated with dysphagia.

**Conclusions:**

The study provides novel information on dysphagia prevalence in patients admitted to an IC unit and its association with geriatric syndromes. Additional research is needed to further define the relationship between geriatric syndromes and dysphagia, and to adequately standardize speech therapist treatments.

**Supplementary Information:**

The online version contains supplementary material available at 10.1007/s40520-025-02950-8.

## Introduction

Dysphagia is relatively common in the general population but the majority of the studies are focused on neurological patients [1]. The prevalence of dysphagia is high among older adults, and the projected ageing of the population makes it an emerging threat for this demographic. The overall prevalence of dysphagia in older persons in the community is about 27% [2]. In the population aged 70 years or older, the prevalence ranges between 27% and 91%, according to the clinical settings, with higher rates observed in acute hospital wards and nursing homes [3]. Despite this high prevalence, dysphagia is not systematically screened for or properly managed in clinical setting, and no research has been conducted in intermediate care (IC) units. IC includes transitional care, that support the needs of older adults, particularly those with chronic disease and frailty, during care transitions and between different settings to enhance recovery, promote ability, optimize management and prevent adverse outcomes including functional decline [4]. 

Dysphagia is recognized as a major geriatric syndrome [5]. The association between dysphagia and other geriatric syndromes is crucial, as these syndromes often overlap and act synergically [6]. The prevalence of impaired nutritional status (including malnutrition risk, and sarcopenia) among older patients with dysphagia, whether associated with either chronic or acute conditions, is high [3, 7]. Frailty is a highly prevalent syndrome in the older patients and is increasingly considered a significant public issue. Among age-related diseases, sarcopenia and dysphagia are two common syndromes in frail older individuals, which can coexist and lead to worse outcomes [8]. In addition to these associations, cognitive impairment also increases the risk of dysphagia. Dysphagia occurs in up to 86% of individuals with advanced dementia; [9] those with cognitive impairment are at a higher risk of developing delirium, and the occurrence of delirium may lead to the onset of dysphagia or worsen a pre-existing diagnosis [10]. However, few studies in acute geriatric and rehabilitation wards have investigated the association between delirium and dysphagia [9, 11]. Finally, a limited number of studies in acute hospital settings, show contrasting evidence of the association between dysphagia and polypharmacy, as well as exposure to specific medications (i.e., benzodiazepines, antipsychotics, opioids) [12–15].

The scarcity of studies investigating the association between dysphagia and other geriatric syndromes in older persons underscores the need for further research, particularly in understudied settings such as IC services. The aims of this study are to report the prevalence of dysphagia among older patients admitted to an IC service, and to investigate its association with geriatric syndromes, including cognitive impairment, delirium, frailty, sarcopenia, malnutrition, and polypharmacy.

## Methods

This is a prospective cohort study of patients 70 years and older admitted between January 2023 and December 2023 to a 78-bed IC unit of the Azienda Speciale Cremona Solidale (Italy). Patients can be admitted to the IC unit either from an acute hospital ward or from the emergency department to facilitate an early supported discharge from the hospital (“step-down”) or from home, to substitute for an episode of inpatient care (“step-up” or admission avoidance pathway) [16]. The study was approved by the ethics committee of the ATS Valpadana, and informed consent was obtained by the patient or by a suitable proxy. Patients were excluded if they had a tracheostomy, were in coma, had severe acquired brain injuries, died, were transferred to an acute hospital during the IC stay, or refused consent to participate in the study. The study was performed in accordance with the principles of the Declaration of Helsinki and reported conforming to the Strengthening the Reporting of Observational Studies in Epidemiology Statement [17]. 

### Data collection and assessment of geriatric syndromes

The data collected at admission included: demographic (age, sex), admission diagnosis, severity of clinical conditions (National Early Warning Score 2, NEWS2), comorbidity (Cumulative Illness Rating Scale, CIRS), delirium (4AT), frailty (Clinical Frailty Scale, CFS), probable sarcopenia (Hand grip), nutritional status (Malnutrition Universal Screening Tool, MUST), number of medications and the medications active on the central nervous system including antipsychotics, benzodiazepines, and opioids.

The NEWS2 score includes respiratory rate, oxygen saturation, need for oxygen therapy, heart rate, blood pressure, level of consciousness/confusion and temperature. The score ranges from 0 to 21 with a score of 0 indicating monitoring every 12 h and a score ≥ 7 requiring continuous monitoring of vital signs [18]. 

CIRS is a comorbidity index, assessing chronic medical illness burden while taking into account the severity of chronic diseases; the score for each of the 14 conditions can range from 1 (absence of pathology) to 5 (maximum level of severity of the disease). The CIRS severity index is the average score of the first 13 items [19]. 

The CFS score ranges from 1 to 9, where 1 indicates a very fit person and 9 a terminally ill individual [20]. The CFS has been widely used in other studies to define the presence of frailty in multiple settings [21]. The CFS score was scored by an expert geriatrician (A.M.), after a multidimensional geriatric assessment [22]. 

The presence of probable sarcopenia was defined according to the European Working Group on Sarcopenia in Older People (EWGSOP2) with a hand grip cut-off < 27 kg for men and < 16 kg for women [23]. Hand grip strength was measured using a non-digital Jamar hydraulic hand dynamometer, as previously described [24]. 

Malnutrition was defined with the MUST scale with a score of 0 indicative of low risk, 1 medium risk and 2 or more high risk of malnutrition [25]. 

Delirium was measured with the 4AT only a subset of 300 patients, since the 4AT was implemented in the clinical evaluation at admission, after June 2023. The 4AT score ranges from 0 to 12 [26]. A 4AT score of 0 suggests the absence of delirium and dementia, a score between 1 and 3 is indicative of possible cognitive impairment but no delirium, and a score ≥ 4 is indicative of delirium. A systematic review reports a high sensitivity (0.88, 95% CI 0.80–0.93) and specificity (0.88, 95% CI 0.82–0.92) of the 4AT for delirium detection in different clinical settings [27]. 

Cognitive function was assessed with the Mini Mental State Examination at admission [28], and the functional status at admission and at discharge with the Barthel Index [29]. 

## Dysphagia assessment and speech therapist treatment

At admission, each patient was assessed by a nurse with the 3-OZ test, followed by a speech therapy consultation to confirm the presence and severity of dysphagia using the DOSS scale. The 3-OZ test is a sensitive screening tool for dysphagia, identifying patients at risk for clinically significant aspiration when the patient shows an adequate level of consciousness and pain control [30]. The DOSS is a seven-point scale developed to rate the functional severity of dysphagia based on objective assessment [31]. The DOSS scale ranges from 7 (normal), 6 (functional limits), 5 (mild dysphagia), 4 (mild to moderate dysphagia), 3 (moderate dysphagia), 2 (moderate-severe dysphagia), 1 (severe dysphagia). The speech therapist (FD) used a clinical evaluation protocol to effectively and efficiently identify patients with possible presbyphagia [32]. At the first and final evaluation (i.e., at the end of treatment) the speech therapist (ST) assessed the patient for the presence of delirium using the 4AT.

ST treatments were categorized as praxis, deglutition, meal type (including observation of eating habits, research into dietary measures such as food consistencies and feeding aids), passive stimulation and counseling.

### Statistical analysis

Continuous variables are presented as mean ± standard deviation, median Interquartile range (IQR) while categorical data as number and proportions. Normal distribution was evaluated for each variable with a pretest for homogeneity of variances. If an abnormal distribution was present, a nonparametric test was used (Mann–Whitney). Otherwise, the Student’s t test for pair comparison was used to examine differences for continuous variables, while the Chi-square test (Fisher’s Test) for categorical variables.

Two multivariable logistic regression models were used to evaluate the association with the presence of dysphagia at admission: Model 1 included age and geriatric syndromes (cognitive impairment, delirium, frailty, probable sarcopenia, malnutrition, polypharmacy); Model 2 included the variables of Model 1 along with the severity of the clinical conditions (NEWS2 score) and the admission diagnosis. For descriptive purposes we described the evolution of dysphagia severity, during the IC stay, according to the presence of the geriatric syndromes.

We conducted an exploratory analysis to investigate the association of dysphagia with delirium in the subset of 300 patients assessed with the 4AT. For descriptive purposes we described the evolution of the presence and severity of dysphagia according to the presence or absence of delirium at the first speech therapist evaluation. We finally explored the association between delirium and dysphagia in a multivariable logistic regression model, including age and other geriatric syndromes.

All statistical analyses were performed using STATA v. 18 (www.stata.com), and statistical significance was set at a p value < 0.05.

## Results

**Fig. 1:**
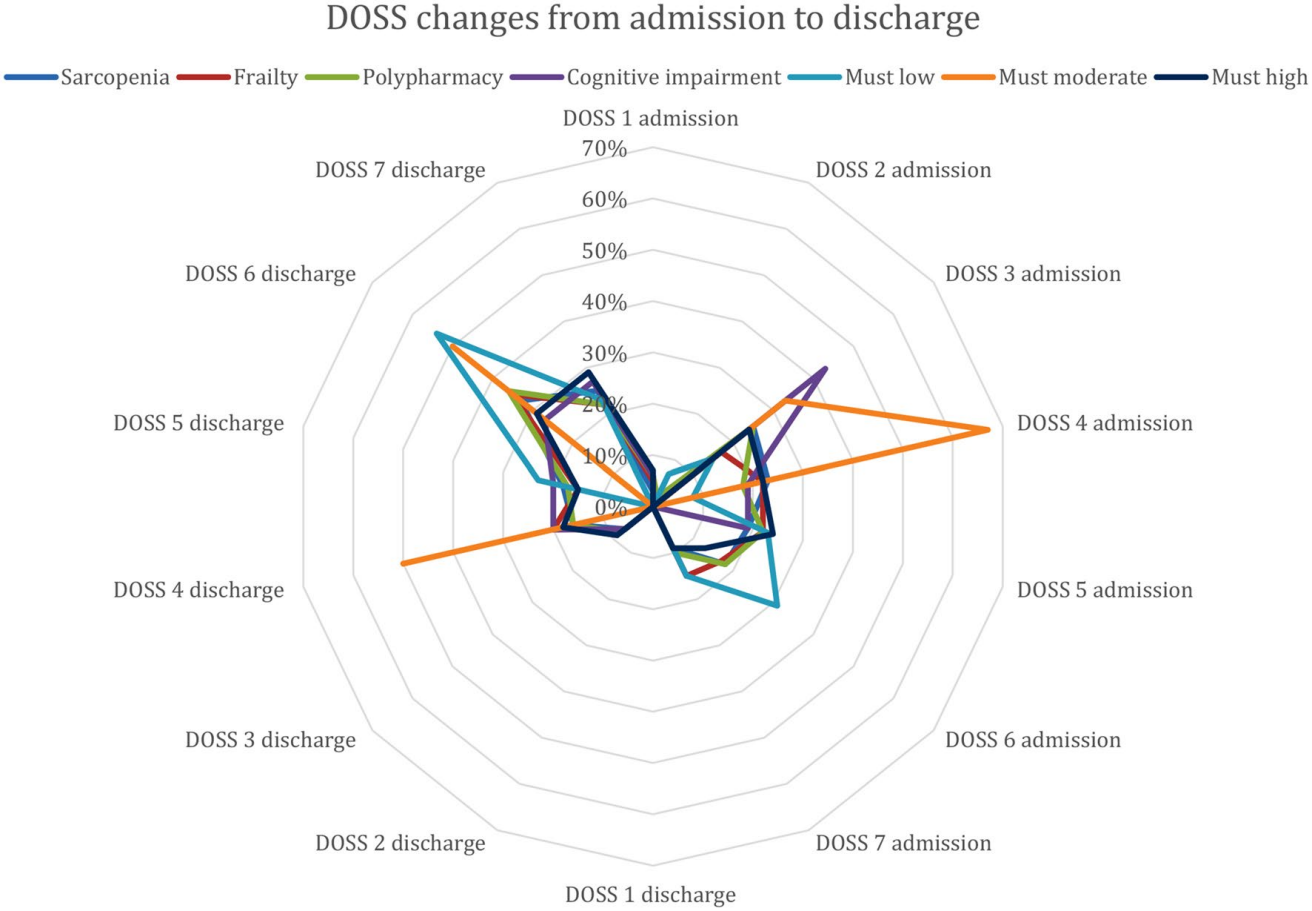
Evolution of the severity of dysphagia, measured with the DOSS scale at admission and at discharge according to the presence of geriatric syndromes evaluated at the time of Intermediate Care admission. *The geriatric syndromes included probable sarcopenia, frailty (Clinical Frailty Scale ≥ 5), polypharmacy (≥ 5 medications), cognitive impairment (Mini Mental State Examination < 24/30), malnutrition risk (Must categories: low, medium, high risk).The severity of dysphagia was measured with the DOSS scale, a seven-point scale developed to rate the functional severity of dysphagia based on objective assessment, with a score from 7 (normal), 6 (functional limits), 5 (mild dysphagia), 4 (mild to moderate dysphagia), 3 (moderate dysphagia), 2 (moderate-severe dysphagia), to 1 (severe dysphagia). In the Figure on the right side is described the severity of dysphagia measured with the DOSS at the time of admission and on the left side at the time of discharge

A total of 455 patients were included in the study. Dysphagia was found in 10% of the patients (Table 1). At admission, 5% of the patients (*N* = 3) had a severe dysphagia (DOSS 1), 32% (*N* = 15) had moderate-severe dysphagia (DOSS 2–3), 23% (*N* = 14) had moderate dysphagia (DOSS 4), 23% (*N* = 14) had mild dysphagia (DOSS 5), 16% (*N* = 10) were within functional limits (DOSS 6), and 10% (*N* = 6) were normal (DOSS 1). At discharge none had severe dysphagia, 7% (*N* = 4) had moderate dysphagia (DOSS 3), 32% (*N* = 20) had mild-moderate dysphagia (DOSS 4–5), 34% (*N* = 21) were within functional limits (DOSS 6), and 26% (*N* = 16) were normal (DOSS 7). Overall, at admission, there was a higher prevalence of mild-moderate dysphagia in patients with cognitive impairment and moderate risk of malnutrition. At discharge, there was still a higher prevalence of mild-moderate dysphagia in patients at moderate risk of malnutrition (Fig. 1).

**Table 1:** Characteristics of the patients according to the presence of dysphagia at admission

Variable	Presence of dysphagia at admission (*N* = 46)	Absence of dysphagia at admission (*N* = 409)	*P* value
Age (years)	85.21 ± 6.82	83.17 ± 8.32	0.05
Gender (female)	28 (61%)	287 (70%)	0.24
CIRS severity	2.11 ± 0.31	1.91 ± 0.31	< 0.01
News2 at admission	1.45 ± 1.89	0.32 ± 0.90	< 0.01
Clinical frailty scale	4.81 ± 2.15	4.38 ± 2.01	0.17
Probable sarcopenia	43 (94%)	3 (6%)	< 0.01
MUST			0.04
Low risk	10 (21%)	154 (38%)	
Moderate risk	4 (2%)	7 (2%)	
High risk	34 (74%)	248 (60%)	
Medications admission	7.47 ± 0.49	7.50 ± 0.15	0.95
Benzodiazepines admission	13 (28%)	97 (23%)	0.47
Antipsychotics admission	10 (22%)	34 (8%)	< 0.01
Opioids admission	3 (6%)	60 (14%)	0.17
Barthel Index admission	14.34 ± 12.72	35.37 ± 25.70	< 0.01
Barthel Index discharge	37.69 ± 25.51	67.79 ± 28.92	< 0.01
MMSE admission	15.89 ± 8.78	21.86 ± 6.05	< 0.01
Setting of admission			0.08
Acute hospital	38 (82%)	302 (74%)	
Home	8 (17%)	107 (26%)	
Admission diagnoses			< 0.01
Neurological	19 (41%)	9 (36%)	
Orthopaedic non elective	9 (20%)	85 (21%)	
Orthopaedic elective	0	43 (15%)	
Pneumological	2 (4%)	27 (4%)	
Cardiological	3 (7%)	27 (6%)	
Gait disturbances	14 (30%)	85 (28%)	

Variables are presented as mean ± standard deviation, median Interquartile range (IQR) while categorical data as number and proportions

**Fig. 2:**
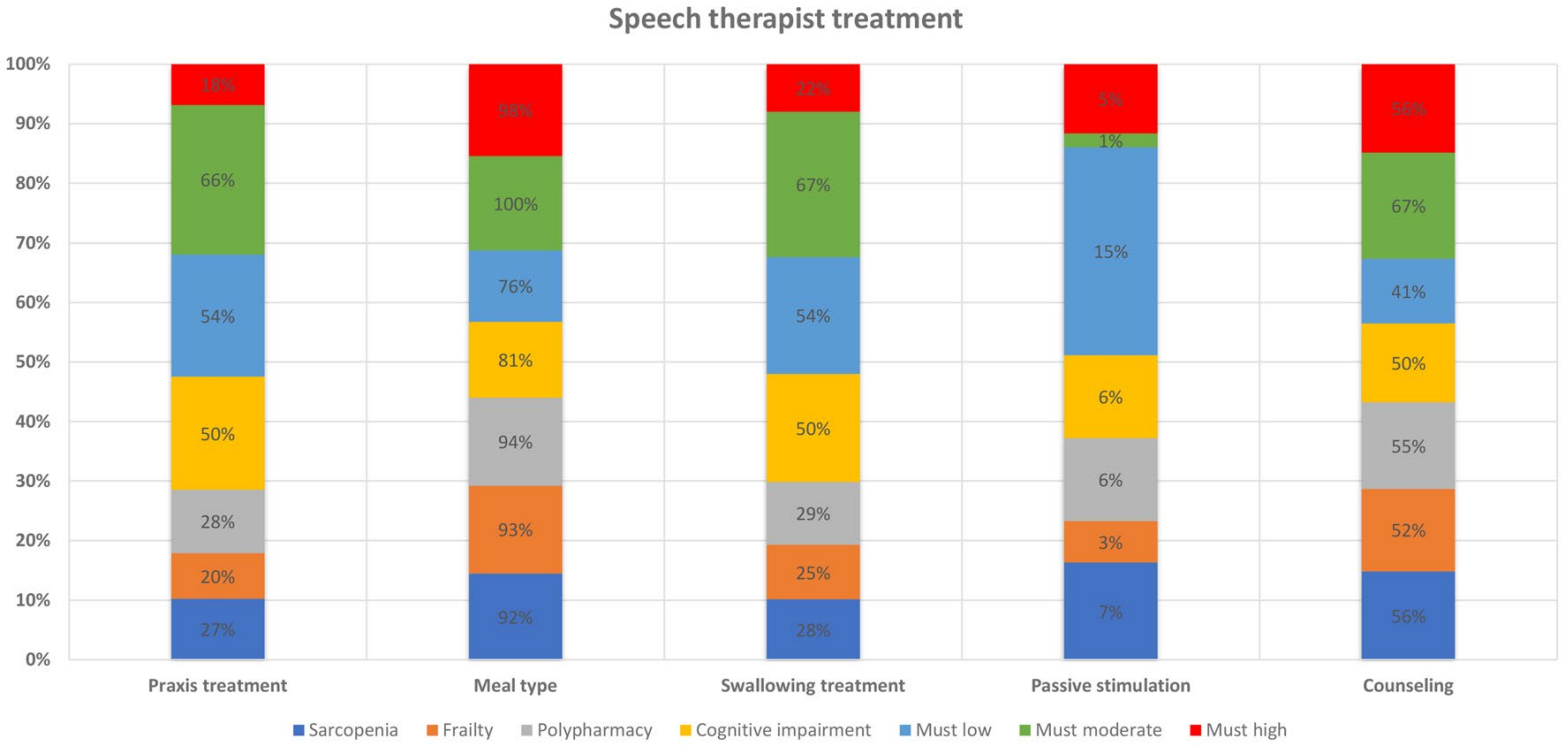
Type of speech therapist treatments according to the presence of geriatric syndromes evaluated at Intermediate Care admission. *The geriatric syndromes included probable sarcopenia, frailty (Clinical Frailty Scale ≥ 5), polypharmacy (≥ 5 medications), cognitive impairment (Mini Mental State Examination < 24/30), malnutrition risk (Must categories: low, medium, high risk)

The most common ST treatment, was the meal treatment (93%), followed by counseling aimed at the patient’s care-givers, nurses and nurses’ aides to share the correct procedures to assist dysphagic patient (53%), swallowing treatment (31%), praxis (27%) and passive stimulation (7%). The mean treatments duration was 388 min, and the mean number of treatments was 10.8 ± 10.8. Praxis treatment was more common in patients with moderate risk of malnutrition Meal treatment and counseling were similar across all geriatric syndromes, while swallowing treatment was more common in patients with cognitive impairment and low-moderate risk of malnutrition (Fig. 2).

A higher prevalence of dysphagia was found in the female population (61%), and those with dysphagia were older (Table 1). The predominant diagnosis in dysphagic patients was related to neurologic disease (41%) followed by gait disturbance due to multiple etiologies (28%) and non-elective orthopedic surgery (20%). The majority of patients in both groups were admitted from acute hospital. Dysphagic patients also had a higher prevalence of probable sarcopenia (94%), moderate to high risk of malnutrition (76%), and higher degree of comorbidity. No difference was detected in the presence of frailty. Patients with dysphagia had a higher severity of clinical conditions. There was no difference in the number of medications, on opioids and benzodiazepines use, while there was a higher prevalence of neuroleptics in those with dysphagia (22% vs. 8%). Dysphagic patients had both greater functional and cognitive impairment compared to non-dysphagic patients, as evidence by lower performance in the Barthel Index and on the MMSE.

In the first multivariable logistic regression model, higher odds of dysphagia were associated with a higher comorbidity (Odds Ratio 6.49, 95% Confidence Interval: 2.02–20.78), and cognitive impairment (OR 0.91, 95% CI: 0.88–10.62) (Table 2). In the second multivariable logistic regression model, higher comorbidity (OR 4.55, 95% CI: 1.18–17.55), cognitive impairment (OR 0.94, 95% CI: 0.88–0.99), and severity of the clinical conditions-NEWS2 (OR 1.61, 95% CI: 1.23–2.13) were associated with higher odds of dysphagia (Table 2). Admission diagnoses, including cardio-pneumological, orthopedic non-elective, and gait disturbances were associated with lower odds of dysphagia compared to neurological diagnoses.

**Table 2:** Multivariable logistic regression model of factors associated with dysphagia at admission

Variable	Model 1			Model 2		
	Odds Ratio	95% Confidence Interval	P value	Odds Ratio	95% Confidence Interval	P value
Age	1.01	0.95–1.07	0.65	1.05	0.98–1.12	0.15
CIRS severity index	6.49	2.02–20.78	**< 0.01**	4.55	1.18–17.55	**0.03**
Mini Mental State Examination ad admission	0.91	0.88–00.96	**< 0.01**	0.94	0.88–0.99	**0.03**
Probable sarcopenia	3.08	0.89–10.62	0.07	1.53	0.42–5.73	0.52
Antipsychotics at admission	2.01	0.76–5.20	0.15	2.23	0.78–6.32	0.13
MUST categories						
Low risk	Ref			Ref		
Medium risk	4.01	0.69–23.30	0.12	3.34	0.48–23.10	0.22
High risk	1.03	0.44–2.39	0.95	0.61	0.23–1.60	0.32
NEWS2 at admission	-			1.61	1.23–2.13	**< 0.01**
Admission diagnoses	-					
Neurological	-			Ref		
Cardio-pneumological	-			1.39	0.33–0.58	**< 0.01**
Orthopaedic elective	-			-		
Orthopaedic elective	-			0.21	0.01–0.71	**< 0.01**
Gait disturbances	-			0.13	0.05–0.36	**< 0.01**

In the exploratory analysis investigating the association between dysphagia and delirium, patients with dysphagia were still older, with higher comorbidity, a higher prevalence of probable sarcopenia, a moderate-high risk of malnutrition. and more severe functional and cognitive impairment (Supplement 1). The prevalence of delirium was higher in dysphagic patients. We did not find a difference in the type of ST treatment in the two groups (Supplement 2). Although the difference was not statistically significant, patients with delirium had lower scores on the DOSS at the first evaluation, indicating greater severity of the dysphagic symptoms. At the end of the ST treatment, all patients were delirium-free, and half of the patients who initially had delirium and medium-severe DOSS scores showed scores indicative of mild dysphagia or no dysphagia. In the multivariable logistic regression (Supplement 3), delirium at admission was associated with higher odds of dysphagia (OR 5.09, CI 1.85–25.01).

## Discussion

The prevalence of dysphagia was 10%, the severity of dysphagia decreased from admission to discharge, with more severe dysphagia found in patients with cognitive impairment and moderate risk of malnutrition. Praxis and swallowing treatments were more common in patients with cognitive impairment and low to moderate risk of malnutrition. Dysphagia was independently associated with higher comorbidity, more severe cognitive impairment, higher clinical instability, and delirium in a subset of patients. However, in the univariate analysis, an association was found between dysphagia and probable sarcopenia, malnutrition, and use of antipsychotics.

The findings of the prevalence of dysphagia in our study aligns with the medical literature, which identifies dysphagia in 10–33% of older adults [33]. In our population, patients with dysphagia had a predominantly neurological initial diagnosis (41%), gait disturbances due to multiple etiologies (30%), non-elective orthopedic surgery (20%). Dysphagia in non-elective orthopedic surgery has been investigated in a few studies, reporting a prevalence ranging from 55 to 77% during the acute phase post-hip fracture [34, 35]. In rehabilitation settings, dysphagia prevalence in elective and non-elective surgery orthopedic patients was 35% [36]. Interestingly, the prevalence of dysphagia in patients with gait disturbances due to multiple etiologies was relatively high (28%). This association should be further explored since these patients might have several leading actors to dysphagia. In the entire sample, we found a higher severity of comorbidity as measured by the CIRS. The CIRS includes different diseases, but we are unable in the current study to investigate the presence and severity of dysphagia according to specific diseases. This analysis might provide insights into the mechanisms underlying the presence of dysphagia, especially in patients with gait disturbance due to multiple etiologies. Previous studies in acute hospital settings confirmed an association between higher comorbidity and dysphagia [15, 37]., also reporting an association with cerebrovascular, ischemic heart, and chronic liver diseases [15]. 

The association between clinical instability and dysphagia is quite novel. A previous large study focused on hospitalized patients with dementia showed a higher prevalence of critical illness in the subgroup of patients with dysphagia [9]. The assessment of clinical conditions with the NEWS2 might indeed be useful if integrated into electronic medical charts, prompting clinicians to evaluate dysphagia if screening is not already integrated in the clinical assessment.

The association between cognitive impairment and dysphagia is well known and it is confirmed in our study [9, 12]. Interestingly, patients with cognitive impairment as those malnourished had a more severe dysphagia at admission and at discharge. Although the association between malnutrition, sarcopenia and dysphagia was only found in univariate analysis, it is important to note that sarcopenia is associated with worse cognitive impairment, and there is a bidirectional relationship between sarcopenia status and cognitive function in older adults [38]. Sarcopenic dysphagia is a complex condition characterized by deglutition impairment due to loss of mass and strength of swallowing muscles, requiring a thorough assessment and management [39]. Additionally, since malnutrition is often associated with cognitive impairment, it has been shown in other studies to be linked to dysphagia. Indeed, guidelines for geriatric assessment support the recommendation to always screen for both, as having severe dysphagia will negatively impact nutritional status and vice versa [40].

Delirium was found to be associated with dysphagia in a subset of patients. This finding confirms the still limited evidence from a few studies on the relationship between these two geriatric syndromes [9, 11]. The presence of delirium and its evolution are elements that influence the patient’s clinical course and rehabilitation possibilities. Delirium alters the ST management, leading to a preference for indirect interventions rather than direct interventions due to the acute cognitive deficits with fluctuations. To date, no studies describe the methods of intervention and care management by ST for patients with delirium, but rather general guidelines, for managing patients with delirium. Given the prevalence of delirium and dysphagia and the clinical complexity they represent, it is necessary to increase interest in the management of these patients to define the methodology of the ST evaluation and treatment.

A lack of association between dysphagia and frailty was observed, which might be related to the scale used to evaluate frailty (i.e. CFS). Previous studies have reported a significant association between frailty and dysphagia in acute hospital settings and community-dwelling persons [8]. Future studies are required to replicate the investigation of the association between frailty and dysphagia, using different tools, such as the Frailty Index.

Finally, we did not find a statistically significant association between dysphagia and polypharmacy, or the use of benzodiazepines and opioids. However, there was an association between the use of antipsychotics at admission and dysphagia in a univariate analysis. Previous studies reported a significant but marginal association between antipsychotics, benzodiazepines, and dysphagia [12–15].

To the best of our knowledge this is the first study to prospectively investigate the prevalence of dysphagia in a heterogenous, large group of patients 70 years and older admitted to an IC, exploring the association with different geriatric syndromes. Limitations of the study include the single-center nature of the study and the evaluation of delirium in a subset of patients.

### Conclusions and implications

The study provides novel information on the prevalence of dysphagia in patients 70 years and older admitted to an IC unit. A significant association was found between dysphagia and two geriatric syndromes (cognitive impairment, delirium, comorbidity). However, the results suggest the necessity to further investigate the relationship with sarcopenia, frailty, and specific drugs that might be implicated in the occurrence of dysphagia (i.e. antipsychotics). The early detection of geriatric syndromes -via a multidimensional and multiprofessional approach- and their clinical manifestations might indeed lead to more favorable outcomes in this population. Additional research is also needed to standardize speech therapist treatment, especially in the context of delirium.

## Electronic supplementary material

Below is the link to the electronic supplementary material.

Supplementary Material 1

Supplementary Material 2

Supplementary Material 3

## Data Availability

No datasets were generated or analysed during the current study.
