# Supplementary material for: Dysphagia and geriatric syndromes in older patients admitted to an intermediate care unit: prospective observational study
Source: Aging Clin Exp Res. 2025 Mar 17;37(1):89. doi: 10.1007/s40520-025-02950-8 (PMC11914323; doi:10.1007/s40520-025-02950-8)
Supplement: Supplementary file 1 — Supplementary Material 1 [file 40520_2025_2950_MOESM1_ESM.docx]

**Supplement 1:** Characteristics of the patients according to the presence of dysphagia at admission including only the subset of patients evaluated for the presence of delirium.

| Variable | Presence of dysphagia at admission  (N= 36) | Absence of dysphagia at admission  (N= 264) | P value |
| --- | --- | --- | --- |
| Age (years) | 85.86 ± 5.72 | 82.98 ± 6.77 | 0.02 |
| Gender (female) | 22 (61%) | 184 (70%) | 0.24 |
| CIRS severity | 2.11 ± 0.29 | 1.89 ± 0.32 | <.01 |
| News-2 at admission | 1.64 ± 1.91 | 0.32 ± 0.92 | <.01 |
| Clinical frailty scale | 4.88 ± 2.10 | 4.38± 2.01 | 0.16 |
| Delirium at admission | 15 (42%) | 30 (11%) | <.01 |
| Probable sarcopenia | 33 (92%) | 3 (8%) | 0.02 |
| MUST |  |  | 0.04 |
| -Low risk | 8 (22%) | 110 (42%) |  |
| -Moderate risk | 2 (6%) | 5 (2%) |  |
| -High risk | 26 (72%) | 149 (56%) |  |
| Medications admission | 7.58 ± 3.62 | 7.46 ± 3.02 | 0.84 |
| Benzodiazepines admission | 13 (36%) | 65 (24%) | 0.16 |
| Neuroleptics admission | 7 (19%) | 21 (8%) | 0.06 |
| Opioids admission | 1 (3%) | 38 (14%) | 0.06 |
| Barthel Index admission | 15.25 ± 13.56 | 35.46 ± 30.03 | <.01 |
| Barthel Index discharge | 39.30 ± 25.42 | 66.69 ± 1.44 | <.01 |
| MMSE admission | 16.56 ± 8.19 | 21.67 ± 6.36 | <.01 |
| Setting of admission |  |  | 0.08 |
| -Acute hospital | 29 (82%) | 193 (73%) |  |
| -Home | 7 (19%) | 71 (27%) |  |
| Admission diagnoses |  |  | <.01 |
| Neurological | 13 (36%) | 24 (9%) |  |
| Orthopaedic fracture | 56 (21%) | 56 (21%) |  |
| Ortopaedic elective | 0 | 33 (12%) |  |
| Pneumological | 2 (6%) | 12 (4%) |  |
| Cardiological | 3 (8%) | 14 (5%) |  |
| Gait disturbances | 10 (28%) | 125 (47%) |  |
