# Supplementary material for: Dysphagia and geriatric syndromes in older patients admitted to an intermediate care unit: prospective observational study
Source: Aging Clin Exp Res. 2025 Mar 17;37(1):89. doi: 10.1007/s40520-025-02950-8 (PMC11914323; doi:10.1007/s40520-025-02950-8)
Supplement: Supplementary file 2 — Supplementary Material 2 [file 40520_2025_2950_MOESM2_ESM.docx]

**Supplement 2:** Evolution of dysphagia and speech therapist treatment according to the presence of delirium, excluding patients not testable for delirium (aphasia, severe hearing loss or visual impairment)

|  | Presence of delirium at the speech therapist evaluation-admission  (N= 15) | Absence of delirium at the speech therapist evaluation-admission  (N=21) | P value |
| --- | --- | --- | --- |
| Severity of dysphagia at the first evaluation, according to the DOSS at admission |  |  | 0.316 |
| DOSS = 1 | 3 (17%) | 0% |  |
| DOSS = 2 | 0 % | 1 (4%) |  |
| DOSS = 3 | 6 (35%) | 5 (17%) |  |
| DOSS = 4 | 3 (17%) | 6 (21%) |  |
| DOSS = 5 | 4 (24%) | 6 (22%) |  |
| DOSS = 6 | 1 (6%) | 4 (14%) |  |
| DOSS = 7 | 0% | 6 (21% |  |
| Severity of dysphagia at the last evaluation, according to the DOSS at discharge |  |  | 0.354 |
| DOSS = 1 | - | - |  |
| DOSS = 2 | - | - |  |
| DOSS = 3 | 2 (12%) | 1 (5%) |  |
| DOSS = 4 | 2 (12%) | 5 (26%) |  |
| DOSS = 5 | 2 (12%) | 5 (26%) |  |
| DOSS = 6 | 9 (53%) | 5 (26%) |  |
| DOSS = 7 | 4 (23%) | 3 (16%) |  |
| Type of treatment |  |  |  |
| -Praxis treatment | 4 (23%) | 9 (32%) | 0.74 |
| -Swallowing treatment | 4 (24%) | 10 (36%) | 0.51 |
| -Meal type treatment | 17 (100%) | 25 (89%) | 0.28 |
| -Passive stimulation treatment | 0 | 0 |  |
| -Counseling | 9 (53%) | 13 (50%) | 1.01 |
| -Time of treatments (minutes) | 303 ± 49 | 431 ± 107 | 0.26 |
| -Number of treatments | 10.47 ± 9.37 | 9.88 ± 28.73 | 0.84 |
