# Supplementary material for: Dysphagia and geriatric syndromes in older patients admitted to an intermediate care unit: prospective observational study
Source: Aging Clin Exp Res. 2025 Mar 17;37(1):89. doi: 10.1007/s40520-025-02950-8 (PMC11914323; doi:10.1007/s40520-025-02950-8)
Supplement: Supplementary file 3 — Supplementary Material 3 [file 40520_2025_2950_MOESM3_ESM.docx]

**Supplement 3:** Multivariable logistic regression model of factors associated with dysphagia at admission including the subset of patients evaluated for the presence of delirium

| Variable | Odds Ratio | 95% Confidence Interval | P value |
| --- | --- | --- | --- |
| Age | 1.06 | 0.99-1.12 | 0.05 |
| CIRS severity | 6.82 | 1.85-25.01 | <.01 |
| Delirium at admission | 5.09 | 2.19-11.80 | <.01 |
| Probable sarcopenia | 1.83 | 0.49-6.88 | 0.36 |
| Must categories |  |  |  |
| Low risk | Ref |  |  |
| Medium risk | 5.40 | 0.79-37.1 | 0.08 |
| High risk | 1.01 | 0.39-2.57 | 0.98 |
